# Convolutional Neural Network for Automatic Identification of Plant Diseases with Limited Data

**DOI:** 10.3390/plants10010028

**Published:** 2020-12-24

**Authors:** Ahmed Afifi, Abdulaziz Alhumam, Amira Abdelwahab

**Affiliations:** 1 Department of Computer Science, College of Computer Science and Information Technology, King Faisal University, P.O. Box 400, Al-Ahsa 31982, Saudi Arabia; aahumam@kfu.edu.sa (A.A.); a.ahmed@kfu.edu.sa (A.A.); 2 Faculty of Computers and Information, Menoufia University, Shibin Al Kawm 32511, Menoufia, Egypt

**Keywords:** crop disease classification, few-shot learning, metric learning, transfer learning

## Abstract

Automated identification of plant diseases is very important for crop protection. Most automated approaches aim to build classification models based on leaf or fruit images. These approaches usually require the collection and annotation of many images, which is difficult and costly process especially in the case of new or rare diseases. Therefore, in this study, we developed and evaluated several methods for identifying plant diseases with little data. Convolutional Neural Networks (CNNs) are used due to their superior ability to transfer learning. Three CNN architectures (ResNet18, ResNet34, and ResNet50) were used to build two baseline models, a Triplet network and a deep adversarial Metric Learning (DAML) approach. These approaches were trained from a large source domain dataset and then tuned to identify new diseases from few images, ranging from 5 to 50 images per disease. The proposed approaches were also evaluated in the case of identifying the disease and plant species together or only if the disease was identified, regardless of the affected plant. The evaluation results demonstrated that a baseline model trained with a large set of source field images can be adapted to classify new diseases from a small number of images. It can also take advantage of the availability of a larger number of images. In addition, by comparing it with metric learning methods, we found that baseline model has better transferability when the source domain images differ from the target domain images significantly or are captured in different conditions. It achieved an accuracy of 99% when the shift from source domain to target domain was small and 81% when that shift was large and outperformed all other competitive approaches.

## 1. Introduction

Since plant diseases may cause crop damage, they pose a major threat to food security and sustainability and may lead to food shortages. Therefore, early detection and control of plant diseases is very important. This process often requires trained human expertise to make the correct diagnosis. However, this expertise is not always available, especially in remote locations and small farms in developing counties. With the advancement of consumer devices, such as smartphones, that can capture high-quality images, the development of an effective image-based diagnostic system can greatly contribute to early disease diagnosis and waste reduction [1]. In the literature, several studies have been proposed to diagnose plant diseases using computer vision, machine learning, or deep learning. In this study we are interested in deep learning-based approaches that have proven effective in many visual recognition tasks.

A number of the techniques that have been proposed focus on diagnosing diseases of a single crop. Esgario et al. [2] proposed an approach to classify and quantify the biotic stress of coffee leaves. They used several Convolutional Neural Network (CNN) models to diagnose and quantify the severity of four coffee plant diseases. They also applied various data augmentation techniques to achieve high diagnostic results. In [3], a deep learning based algorithm for image-based detection of cassava plant disease has been proposed. The authors applied the transfer learning of a pretrained CNN model to train a linear classifier, support vector machine (SVM), and a nearest neighbor (KNN) classifier using a dataset of cassava disease images captured in the field. Their best model achieved an average accuracy of 93% for three diseases and two types of pest damage. A similar approach has also been proposed for detection and diagnosis of cassava plant diseases in [4]. The tomato crop has attracted the interest of many researchers and several techniques have been proposed to diagnose its diseases [5,6,7]. An approach for diagnosing corn leaf diseases has been proposed in [8]. A dataset collected with a smartphone camera was used to train a custom CNN model and a good accuracy was obtained using a limited number of test images. Likewise, approaches have been proposed using CNNs trained from scratch or using transfer learning to classify apple plant diseases [9,10], grape plant diseases [11], and potato plant diseases [12].

Another class of methods aims to provide general solutions that can be used to diagnose several diseases simultaneously. Too et al. [13] performed a comparative study to fine-tune several CNN models for identifying plant diseases. The results of this study using the PlantVillage dataset [14] indicated that DenseNet121 [15] outperformed all other models and achieved an accuracy score of 99.75%. $PD^{2}SE-Net$ was proposed in [16], based on ResNet50 [17], to identify plant species, diagnose diseases, and estimate disease severity. This approach achieved an accuracy score of 98% for plant disease classification. Many similar methods of classifying multicrop diseases have been also proposed in the literature as presented in [18,19]. Lee et al. [20] approached the problem differently, by comparing a number of techniques for classifying plant diseases based on the common disease name. This formulation allowed them to build a more generalizable model. In this study, we will also evaluate this formulation in the case of learning from little data.

If we look at the literature, we can realize the effectiveness of deep learning methods for diagnosing plant diseases. However, on the other hand, the success of these techniques depends greatly on the availability of a good amount of data to train the models, and the lack of data may hinder the development of these techniques. This data collection and labeling are often challenging, either because of the novelty of the disease or because of the high cost or lack of appropriate expertise. Therefore, the focus is on learning techniques that can learn from small datasets, formally called few-shot learning (FSL) [21,22,23]. There is a variety of FSL learning approaches that can be organized into four main categories: metalearning, metric learning, data augmentation, and transfer learning. Algorithms in metalearning category learn a learning strategy or a good model initialization to adjust well to a novel, few-shot learning task [24,25,26]. The metric learning approaches learn a semantic embedding space using a distance loss function. Accordingly, they map images in a space where similar classes are close together while different classes are further apart. Metric learning is an active area of research in which several algorithms have been proposed such as Siamese network [27], Triplet network [28], matching network [29], prototypical network [30], and relation network [31]. The data augmentation approaches generate more data from novel classes to facilitate the regular learning [32,33]. This area of research is under active development and there are a number of studies that have focused on comparing different FSL methods. Chen et al. [23] conducted a study to compare several metalearning, metric learning, and transfer learning methods using natural images. In another recent study [34], few-shot learning methods were compared when there is a shift between source and target domains. These studies showed that the performance of few-shot learning approaches may vary depending on the source of the images and the problem formulation. It also indicated that few-shot learning techniques based on metalearning poorly perform when there is a significant shift between source and target domains.

Recently, some FSL-based approaches have been proposed for identifying plant species and diagnosing crop diseases. Hu et al. [35] generate synthetic leaf images to facilitate tea leaves disease identification. The Siamese network was used in [36] to build a classification approach for diagnosing citrus diseases and in [37] to build a plant leaf classification approach. In [38], Argueso et al. introduced an enhanced approach to classify plant leaf diseases through the use of Triplet loss [28] and support vector machines (SVM) [39]. They created the source and target domains datasets from the PlantVillage [14] dataset and fine-tuned the last fifty layers of the Inception V3.0 model. Their results using the Triplet loss to create an embedding space and SVM classifier for FSL outperformed the fine-tuned model. However, these results were influenced by their fine-tuning setup because it is difficult to optimize a large number of parameters with just a few images.

**Figure 1:**
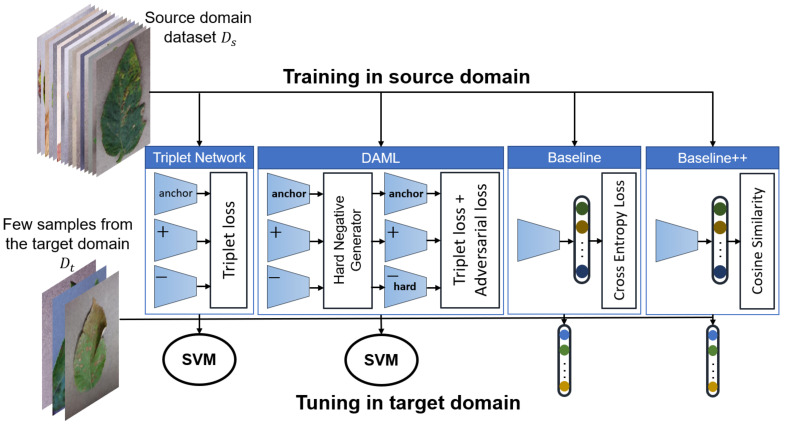
Block diagram of the developed plant disease classification approaches.

Since selection of an appropriate learning strategy and models, as well as the right formulation of the problem, are key factors to the success of the FSL approach, the main purpose of this study is to provide an effective plant disease diagnosis approach that can learn from little data. The contributions of this study can be summarized as follows:

1. Development and evaluation of several FSL approaches to classify plant diseases based on Triplet network, Deep Adversarial Metric Learning (DAML) [40] and transfer learning using both linear and cosine-similarity classifiers [23]. All approaches were evaluated using images captured under the same as well as different conditions. We have found that fine-tuning of a pretrained model outperforms all other approaches. Figure 1 shows an overview of the approaches developed in this study.
2. We investigate the impact of model complexity on the performance of the FSL approaches, ResNet18, ResNet34, and ResNet50 were used in this study. The results showed that fine-tuning using linear classifiers benefits from increased model complexity while this cannot be confirmed for other approaches.
3. We examined two different formulations in this study. In the first, we classify plants and diseases together, while in the second we focus on diseases only. The second formulation helped the model to achieve better results in identifying new diseases and to better adapt to images captured under different conditions.

## 2. Materials and Methods

### 2.1. Datasets

Two datasets were used for training and evaluating all few-shot learning algorithms in this study, namely the PlantVillage dataset [14] and the coffee leaf dataset [2]. PlantVillage dataset has 54,305 leaf images of 14 crop species and 26 diseases distributed among 38 crop-disease pairs. This dataset contains clear images of plant leaves and each image contains only one leaf. It also comes with preset training/testing subsets which we follow in this study. Table 1 gives a summary of these classes and the number of images in each class. We use this dataset in two different ways, first to classify disease-crop pairs and second to classify disease regardless of the affected crop. In the first configuration, 32 classes (C7 to C38) are used as the source domain $D_{s}$ and 6 classes (C1 to C6) as the target domain $D_{t}$ similar to [38]. In this case the source domain has 43,444 samples while the target/novel domain has 10,861 samples. In the second configuration, we rearrange the dataset according to the common disease name as in [20], resulting in 20 disease classes and one healthy class. For diseases that affect more than one plant, all samples are combined in one class under the name of this disease, and for the healthy class, about 5000 samples were collected from all available plants. The summery of the rearranged dataset is shown in Table 2. The three diseases with the fewest images (CD1, CD4, and CD19) were selected from the rearranged dataset as target (novel) domain classes and the rest as the source domain classes. The number of samples in the source domain $D_{s}$ is 44,081 and in the target domain $D_{t}$ is 1278.

**Figure 2:**
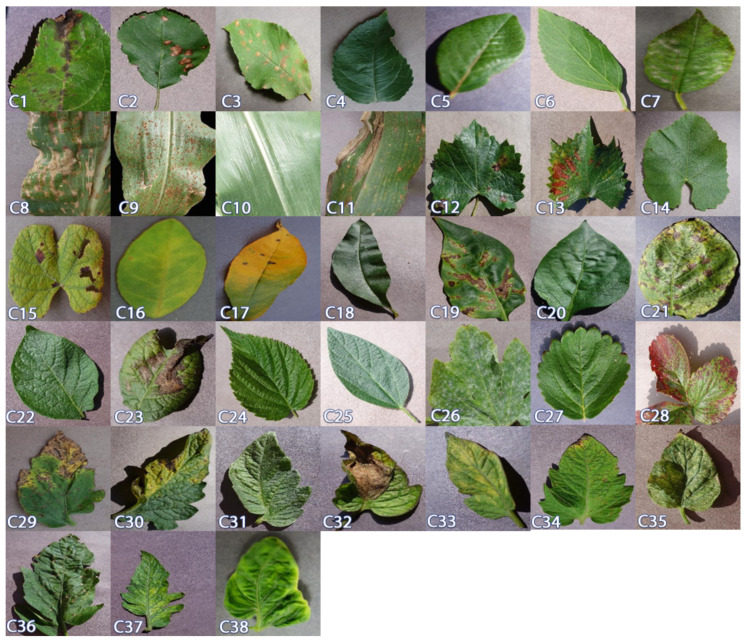
Sample images from PlantVillage dataset. For the details of different classes, check Table 1.

**Figure 3:**
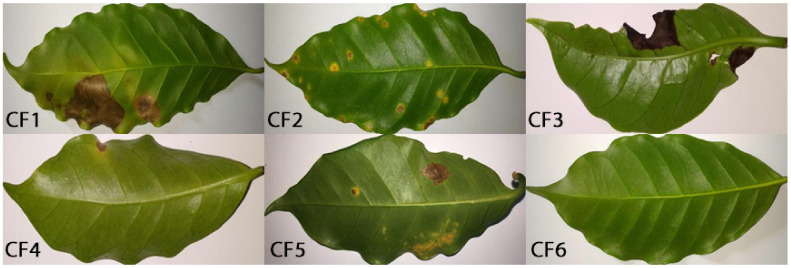
Sample images from coffee leaf dataset. For the details of different classes, check Table 3.

The coffee leaves Dataset [2] contains 1747 Images of Arabica coffee leaves captured using different mobile phones. It contains healthy leaves as well as leaves affected by one or more diseases (leaf miner, rust, brown leaf spot, cercospora leaf spot). Each leaf is labeled with the predominant disease. In this study, we did not use this dataset for training and all classes were used as target/novel domain classes. This configuration allows us to fairly evaluate the performance of the developed models in a more realistic situation, when the novel dataset is captured under different condition. The summary of this dataset’s classes is presented in Table 3. Samples from PlantVillage and coffee leaf datasets are shown in Figure 2 and Figure 3, respectively.

### 2.2. Learning Approaches

In this study, we aim to develop an approach that can learn from little data. This problem in the community is known as few-shot learning (FSL), which can leverage the information learned from a large source domain dataset, $D_{s}$, to build a model that can classify novel classes from target domain, $D_{t}$, using few samples. In FSL, the target domain has a label space that differs from the source domain and it is formally defined as C−way/K−shots classification problem; Classify *C* novel classes using *K* sample from each class. The idea behind all FSL algorithms is to build a generic feature extractor or embedder, *f*, to map the image $X_{i}$ to a low-dimensional feature vector $f_{i}=f\left(X_{i},\theta \right)$, θ is the embedder parameters. This embedding trained from source data, $D_{s}$, should be general enough to be used for classifying new classes in the target domain, $D_{t}$. Since the classification of plant diseases differs from the classification of digits or general objects, we will develop several classification models using little data and compare them to build our final model. Details of learning algorithms and methodologies will be presented in the following subsections.

### 2.3. Transfer Learning: Baseline and Baseline++

**Figure 4:**
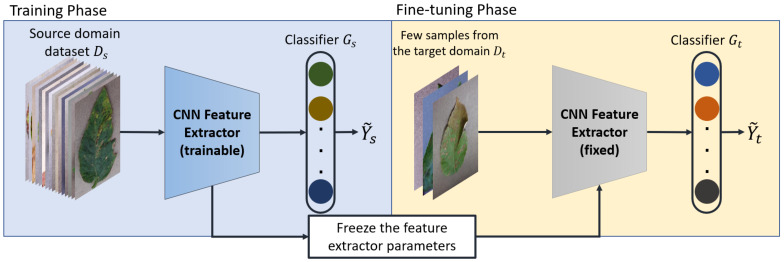
An overview of the transfer learning methodology.

In transfer learning, a CNN model is trained using the source domain dataset and then fine-tuned using few samples from the target domain dataset as shown in Figure 4. In the training phase, both the feature extractor *f* and the classifier $G_{s}$ are trained from scratch while in the fine-tuning phase, the feature extractor is fixed and a new classifier $G_{t}$ is trained using a small target domain dataset. Similar to [23], in this study, we use two baseline models. The first uses a linear classifier and we refer to it as Baseline and the other uses a cosine-similarity based classifier and we refer to it as Baseline++.

The Baseline model uses a linear classifier $G_{s}\left(.|W_{s}\right)$, $W_{s}\in R^{d\times c}$ is the weight matrix, *d* is the dimension of the feature vector and *c* is the number of classes. This classifier has a linear layer followed by a Softmax function σ as defined in Equation (1).

(1) $$\tilde{Y}=\sigma (W_{s}^{T}f\left(X_{i},\theta \right))$$

In the Baseline++ classifier, the weight matrix $W_{s}$ consists of *c*, *d*-dimensional weight vectors $\left[w_{s1},w_{s2},\ldots ,w_{sc}\right]$. Each weight vector, $w_{sj}$, can be considered as a prototype for one class. During the training process, the cosine similarity $cs_{ij}$ between the feature vector $f_{i}$ and the weight vector $w_{s}^{j}$ is calculated as in Equation (2) and the final classification probability is obtained by normalizing the similarity vector, $\left[cs_{i1},cs_{i2},\ldots ,cs_{ic}\right]$ using a Softmax function. In this study, we use cross-entropy loss to train and fine-tune both the Baseline and Baseline++ models.

(2) $$cs_{ij}=\frac{f{\left(X_{i},\theta \right)}^{T}w_{sj}}{\|f\left(X_{i},\theta \right)\left\|\right\|w_{sj}\|}$$

### 2.4. Metric Learning Using Triplet Network

**Figure 5:**
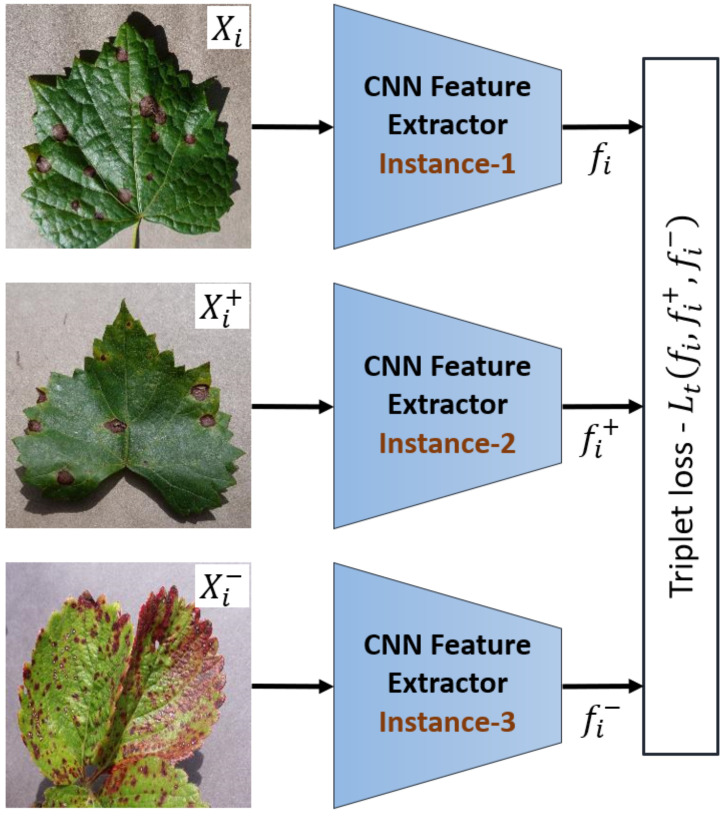
The structure of the Triplet network, the three instances have the shared weights.

The Triplet network [28] uses three instances of the same feature embedder with shared weights to embed the input triplet which contains an anchor sample $X_{i}$, a positive sample $X_{i}^{+}$ and a negative sample $X_{i}^{-}$. Anchor and positive samples are sampled from the same class while the negative sample is sampled from a different class. This network is trained from scratch using the source domain dataset, $D_{s}$, by minimizing the triplet loss function [41] defined in Equation (3). The structure of the Triplet network is shown in Figure 5. During the training phase, the network reduces the distance between the anchor and its positive pair while increasing the distance to the negative sample.

(3) $$L_{t}\left(f_{i},f_{i}^{+},f_{i}^{-}\right)=max\left(0,D{\left(f_{i},f_{i}^{+}\right)}^{2}-D{\left(f_{i},f_{i}^{-}\right)}^{2}+m\right),$$

where, $f_{i}$, $f_{i}^{+}$ and $f_{i}^{-}$ are the feature vectors corresponding to $X_{i}$, $X_{i}^{+}$ and $X_{i}^{-}$, respectively. D(.,.) is the Euclidean distance and *m* is the margin.

After training from source domain dataset, to classify plant diseases with little data, a metric learning approach based on [22] is utilized. In this approach, the trained embedder is used to extract the features of few target domain samples, $D_{t}$, and a multiclass Support Vector Machine (SVM) [39] classifier is used for final classification. SVM solves the classification problem by finding a set of hyper planes in the d−dimensional space that separates samples from different classes. It tries to find the planes with the maximum margin, or distance, from data points on both sides. In this study, one versus all multiclass SVM classifier with linear kernel was used.

### 2.5. Deep Adversarial Metric Learning (DAML)

DAML [40] attempts to improve traditional metric learning (using triplet loss) by generating synthetic hard negatives from easy negatives. This allows the learning algorithm to take advantage of the large number of easy negatives and increases the diversity and representation of negative samples near the margin. It jointly trains a hard negative generator and a distance metric by minimizing the loss function defined in Equation (4).

(4) $$J\left(f_{i},f_{i}^{+},f_{i}^{-}\right)=J_{gen}\left(f_{i},f_{i}^{+},f_{i}^{-}\right)+\lambda J_{m}\left(f_{i},f_{i}^{+},f_{i}^{-}\right),$$

where $f_{i},f_{i}^{+},f_{i}^{-}$ are the feature vectors corresponding to the anchor, positive and negative samples and λ parameter represents the balance between the metric loss $J_{m}$ and the adversarial loss $J_{gen}$. The generator receives triplets $\left(f_{i},f_{i}^{+},f_{i}^{-}\right)$ and generates hard negatives ${\tilde{f}}_{i}^{-}$ and it is trained by minimizing the objective function defined in Equation (5). Accordingly, the generator aims to create negative images close to the anchor image and similar to other negatives and at the same time may fool the metric- learning. The triplet loss defined in Equation (6) is utilized here for metric learning. In this study, three-layer fully connected network was used as a feature generator and the input to the generator is a combination of anchor, positive and negative samples and the output is a synthetic hard negative.

(5) $$\begin{array}{c} \begin{array}{cc} J_{gen}\left(f_{i},f_{i}^{+},f_{i}^{-}\right) & =\;J_{hard}+\lambda_{1}J_{reg}+\lambda_{2}J_{adv}\\ \; & =\;\|{\tilde{f}}_{i}^{-}-f_{i}{\|}_{2}^{2}+\;\lambda_{1}{\left\|{\tilde{f}}_{i}^{-}-f_{i}^{-}\right\|}_{2}^{2}\\ \; & +\lambda_{2}max\left(0,D{\left(f_{i},{\tilde{f}}_{i}^{-}\right)}^{2}-D{\left(f_{i},f_{i}^{+}\right)}^{2}-\alpha \right), \end{array} \end{array}$$

$\lambda_{1}$ and $\lambda_{2}$ are two balancing parameters.

(6) $$J_{m}\left(f_{i},f_{i}^{+},f_{i}^{-}\right)=max\left(0,D{\left(f_{i},f_{i}^{+}\right)}^{2}-D{\left(f_{i},{\tilde{f}}_{i}^{-}\right)}^{2}+m\right),$$

Similar to the metric-learning using Triplet network, feature embedder and generator were trained with the source domain data and a multiclass SVM was used for FSL in the target domain.

### 2.6. Deep Architectures and Experimental Setup

In all experiments, we used three CNN models having different complexities, namely, ResNet18, ResNet34, and ResNet50 [17]. We use models of the same architecture (Deep residual learning) but with different complexity to study the impact of model complexity on the classification accuracy. The main idea behind deep residual learning is to use identity shortcut connections to force each network block to fit a residual mapping H(X)=F(X)+X, where *X* is the input feature and F(X) is the output of this block. This formulation, as demonstrated by the original authors, can solve the problem of vanishing/exploding gradient and alleviate accuracy degradation in deep networks. All residual architectures share the same structure but have different number of blocks and layers. ResNet18 has about 12 million trainable parameters and an output size of 512, ResNet34 has about 21 million trainable parameters and an output size of 512 and ResNet50 has about 26 million trainable parameters and an output size of 2048. For metric learning, we use a fully connected layer with ReLU activation function to map the backbone output to a 128-dimensional vector.

All models were trained using the entire source domain dataset, after that each model was fine-tuned using a small set of target domain images ranging from 5 to 50 in increments of 5. Baseline and Baseline++ models were trained in source domain using Adam optimizer [42] with a learning rate of 0.0001 for 30 epochs and the best model was used for the fine-tuning phase. For metric learning using triplet loss, a margin of 1 was used in the loss function and the model was trained using Adam optimizer with a learning rate of 0.0001 for 15 epochs. The Deep Adversarial Metric Learning used Adam optimizer for backbone model and generator optimization with a learning rate of 0.0001 and a margin of 1 for the triplet loss. The training dataset was augmented using random horizontal and vertical flipping, rotation in a range of 0 to 30, color change (hue, saturation, brightness and contrast) by a factor of 0.1 and center cropping.

In target domain, the baseline and baseline++ models were fine-tuned using Stochastic Gradient Decent (SGD) optimizer with a learning rate of 0.01, momentum of 0.9, and weigh decay of 0.001 for 100 epochs. SVM with linear kernel was used for triplet loss and DAML methods and the soft margin parameter was set to C=1. All experiments were repeated 100 times and the average accuracy was calculated using a query set of 50 images per class. All experiments were conducted using PyTorch [43] deep learning framework.

## 3. Results and Discussion

In this study, the performance of four learning approaches and three backbone models for classifying crop diseases from limited data was evaluated. We use two different formulations; one considers both the classification of crops and diseases as a single task as [38] and the other focuses on the classification of diseases only using the common disease name as recommended by [20]. The results of all experiments will be presented in this section.

### 3.1. Crop and Disease Classification

**Figure 6:**
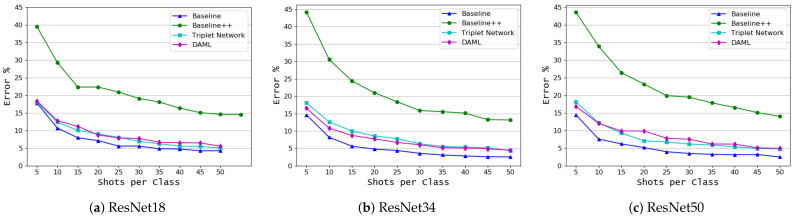
Classification based on both crop and disease types (PlantVillage target domain dataset), mean error as a function of the number of shots per class, *K*, for 4 learning algorithms and 3 backbone models.

In this set of experiments, 32 classes of the PlantVillage dataset [14] were used as the source domain data, $D_{s}$, as detailed previously. Two datasets with different characteristics were used as the target domain data. The first one contains six different classes of the PlantVillage dataset and the other is the coffee leaf dataset [2] which contains six classes of coffee plant diseases and was collected under different conditions from the source domain dataset. Figure 6 shows the results for the first target domain dataset. This figure clearly indicates that the Baseline model outperforms all other learning approaches with all backbone models and for all few-shot settings. It achieved the lowest error rate of 2.48% for 50 shots and 14.42% for five shots with the ResNet50 backbone model slightly outperforming the ResNet34 backbone model. DAML comes in the second place, with a lower error rate than Triplet network for most of the few-shot settings especially when the number of samples is very low. Both approaches work better with the ResNet34, which is slightly outperformed the ResNet50. The Baseline++ lags behind other approaches significantly. The results of these experiments are summarized in Table 4.

**Figure 7:**
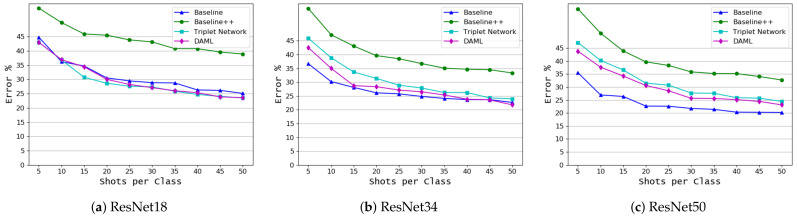
Classification based on both crop and disease types (coffee leaf [2] target domain dataset), mean error as a function of the number of shots per class, *K*, for 4 learning algorithms and 3 backbone models.

The results of the coffee leaf dataset are shown in Figure 7 and Table 5. These results are consistent with the previous results, as the Baseline model achieved the lowest error rate of 20.22 for 50 shots and 35.54 for five shots with the ResNet50 backbone model and it outperformed all other approaches. DAML, with ResNet50, is second, while Baseline++ is the last. This figure also indicates that the large shift between the source domain and the target domain data leads to some degradation in performance.

### 3.2. Disease Classification Based on Common Disease Name

Since the same disease may affect more than one crop, the focus of these experiments will be on classifying diseases regardless of which plant is affected. This formulation is more realistic in the real world, where the farmer knows which crops he is growing but wants to learn about the diseases that afflict them. Eighteen classes of the rearranged PlantVillage dataset, as in Table 2, were used as the source domain dataset. Three new diseases from the PlantVillage dataset were used as the first target domain dataset and coffee leaf dataset as the second target domain dataset.

**Figure 8:**
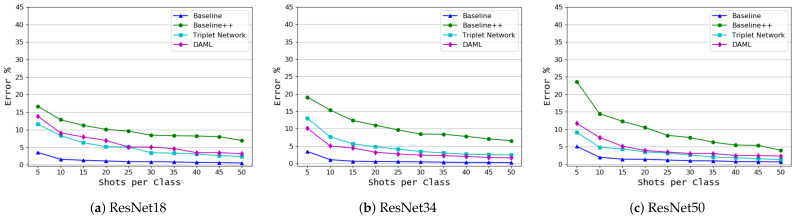
Classification based on the common disease name (rearranged PlantVillage target domain dataset—see Table 2), mean error as a function of the number of shots per class, *K*, for 4 learning algorithms and 3 backbone models.

**Figure 9:**
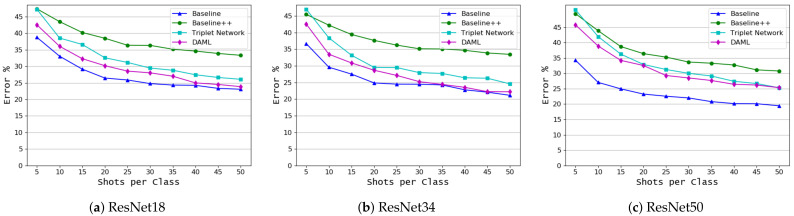
Classification based on the common disease name (coffee leaf [2] target domain dataset, mean error as a function of the number of shots per class, *K*, for 4 learning algorithms and 3 backbone models.

The results shown in Figure 8 for the first target domain dataset (based on disease name) indicate that the baseline model outperformed all other approaches. It achieved a very low error rate even with a very few shots. The DAML and Triplet network exchange are second place based on the number of samples and the backbone model, but they fall behind the baseline model by a large margin, especially in the case of very few shots. The summary of these results is shown in Table 6. For the coffee leaf dataset, the classification results based on the disease name are shown in Figure 9. Here, we can see the consistent performance of the baseline model and the DAML approach. Baseline model achieved the lowest error rate of 19.44 for 50 shots and 34.28 for 5 shots while DAML achieved an error rate of 22.2 for 50 shots and 42.6 for 5 shots. Table 7 shows the classification results for this dataset.

The results presented in this study show that we can use a good baseline model trained from a large source domain dataset to build a model that can learn from little data. Compared to the other approach, the baseline model can take advantage of the complexity of the backbone model to build a good representation of the features. Choosing the right backbone model and learning strategy is also important. Formulating the problem based on the name of the common disease can lead to better and more consistent results and enhances generalization of the model.

In a previous study [38], Argueso et al. indicated that metric learning using triplet loss outperformed the transfer learning strategy by a large margin. However, in their study they have tuned 50 Inception V3 [44] layers which cannot be achieved with a few samples and we believe that this is the main reason this model fails. Table 8 shows the comparison of the baseline model, the triple network with the ResNet50 backbone that was used in this study and the work of Argueso et al. [38]. This comparison shows that although a ResNet50 backbone trained from scratch using source domain data enhances Triplet network performance, the baseline model outperforms all other approaches. This is due to the fact that many diseases affect a small portion of the plant leaf, which hinders accurate representation of these diseases in the embedding space of the metric learning techniques. However, the baseline model directly extracts the features that allow it to distinguish different diseases, especially in the second formulation.

## 4. Conclusions

In this study, we developed several approaches for classifying plant diseases that can learn from little data. Transfer learning, Triplet network, and Deep Adversarial Metric learning (DAML) were used to build these approaches. The evaluation of these approaches demonstrated the efficiency of transfer learning using a good baseline model. It achieved a very high accuracy of 99% for new classes when the source and target domain data are captured under the same condition and a reasonable accuracy of 81% for novel dataset that is captured under different conditions. It can generalize well and beat all competitive approaches. We also found that DAML can enhance the traditional metric learning by generating hard samples and increases the data diversity. Selection of the appropriate model and learning strategy is also essential to the success of the FSL approach. Therefore, developing a disease classification method regardless of the plant can perform better and it is more appropriate for real applications than the usual method, which is to classify both disease and plant type together. Finally, we believe that focusing only on the affected part of the plant might lead to better classification results, especially for diseases that affect a small portion of the leaf.

## Figures and Tables

**Table 1:** Summary of the PlantVillage datase [14].

Class	Crop	Disease	Samples	
			Train	Test
C1	Apple	Apple cab	504	126
C2	Apple	Black rot	496	125
C3	Apple	Cedar apple rust	220	55
C4	Apple	Healthy	1316	329
C5	Blueberry	Healthy	1202	300
C6	Cherry (including sour)	Healthy	684	170
C7	Cherry (including sour)	Powdery mildew	842	210
C8	Corn (maize)	Cercospora leaf spot Gray leaf spot	410	103
C9	Corn (maize)	Common rust	953	239
C10	Corn (maize)	Healthy	929	233
C11	Corn (maize)	Northern Leaf Blight	788	197
C12	Grape	Black rot	944	236
C13	Grape	Esca (Black Measles)	1107	276
C14	Grape	Healthy	339	84
C15	Grape	Leaf blight (Isariopsis Leaf Spot)	861	215
C16	Orange	Haunglongbing (Citrus greening)	4405	1102
C17	Peach	Bacterial spot	1838	459
C18	Peach	Healthy	288	72
C19	Pepper bell	Bacterial spot	797	200
C20	Pepper bell	Healthy	1183	295
C21	Potato	Early blight	800	200
C22	Potato	Healthy	121	31
C23	Potato	Late blight	800	200
C24	Raspberry	Healthy	297	74
C25	Soybean	Healthy	4072	1018
C26	Squash	Powdery mildew	1468	367
C27	Strawberry	Healthy	364	92
C28	Strawberry	Leaf scorch	887	222
C29	Tomato	Bacterial spot	1702	425
C30	Tomato	Early blight	800	200
C31	Tomato	Healthy	1273	318
C32	Tomato	Late blight	1527	382
C33	Tomato	Leaf mold	761	191
C34	Tomato	Septoria leaf spot	1417	354
C35	Tomato	Spider mites Two-spotted spider mite	1341	335
C36	Tomato	Target spot	1123	281
C37	Tomato	Tomato mosaic virus	299	74
C38	Tomato	Tomato Yellow Leaf Curl Virus	4286	1071

**Table 2:** Summary of the PlantVillage dataset [14] based on disease name.

Class	Disease	Affected Plants	Samples	
			Train	Test
CD1	Apple scab	Apple	504	126
CD2	Bacterial spot	Peach, Pepper bell, Tomato	4337	1084
CD3	Black rot	Apple, Grape	1140	361
CD4	Cedar apple rust	Apple	220	55
CD5	Cercospora leaf spot Gray leaf spot	corn	440	103
CD6	Common rust	corn	953	239
CD7	Early blight	Potato, Tomato	1600	400
CD8	Esca black measles	Grape	1107	276
CD9	Haunglongbing Citrus greening	Orange	4405	1102
CD10	Late blight	Potato, Tomato	2327	582
CD11	Leaf blight Isariopsis Leaf Spot	Grape	861	215
CD12	Leaf mold	Tomato	761	191
CD13	Leaf scorch	Strawberry	887	222
CD14	Northern Leaf blight	Corn	817	197
CD15	Powdery mildew	Cherry, Squash	2310	577
CD16	Septoria leaf spot	Tomato	1417	354
CD17	Spider mites Two spotted spider mite	Tomato	1341	335
CD18	Target spot	Tomato	1123	281
CD19	Tomato mosaic virus	Tomato	299	74
CD20	Tomato Yellow Leaf Curl Virus	Tomato	4286	1071
CD21	Healthy	−	4909	1200

**Table 3:** Summary of the Coffee Leaf dataset [2].

Class	Predominant Disease	Samples
CF1	Leaf miner	387
CF2	Rust	531
CF3	Brown leaf spot	348
CF4	Cercospora leaf spot	147
CF5	(Several with same severity)	62
CF6	Healthy	272

**Table 4:** Classification based on both crop and disease types (PlantVillage target domain dataset), the lowest error for 5, 25, and 50 shots is highlighted in bold.

Approach	ResNet18/(Shots)			ResNet34/(Shots)			ResNet50/(Shots)		
	5	25	50	5	25	50	5	25	50
Baseline	17.78	5.58	4.24	14.6	4.38	2.52	**14.42**	**3.98**	**2.48**
Baseline++	39.48	20.92	14.58	44.22	18.4	13.16	43.62	19.92	14.02
Triplet Network	18.06	8.04	5.06	18.1	7.8	4.28	18.14	6.74	4.8
DAML	18.38	7.9	5.52	16.54	6.7	4.46	16.84	7.82	4.92

**Table 5:** Classification based on both crop and disease types (Coffee leaf [2] target domain dataset), the lowest error for 5, 25, and 50 shots is highlighted in bold.

Approach	ResNet18/(Shots)			ResNet34/(Shots)			ResNet50/(Shots)		
	5	25	50	5	25	50	5	25	50
Baseline	44.78	29.46	25.08	36.68	25.76	22.66	**35.54**	**22.62**	**20.22**
Baseline++	54.96	43.84	38.38	56.7	38.5	33.32	60.12	38.32	32.7
Triplet Network	43.02	27.6	23.5	45.9	28.9	23.96	47.14	30.74	24.48
DAML	43	28.2	23.58	42.52	27.14	21.78	43.82	28.58	23.14

**Table 6:** Classification based on the common disease name (rearranged PlantVillage target domain dataset—see Table 2), the lowest error for 5, 25, and 50 shots is highlighted in bold.

Approach	ResNet18/(Shots)			ResNet34/(Shots)			ResNet50/(Shots)		
	5	25	50	5	25	50	5	25	50
Baseline	**2.52**	0.9	0.52	3.46	**0.56**	**0.28**	5.08	1.018	0.66
Baseline++	16.7	9.6	6.94	19.08	9.68	6.56	23.58	8.24	3.98
Triplet Network	11.64	5.06	2.4	13.04	4.14	2.48	9.1	3.18	1.32
DAML	13.84	5.1	3.18	10.18	2.74	1.62	11.66	3.44	2.32

**Table 7:** Classification based on the common disease name (coffee leaf [2] target domain dataset), the lowest error for 5, 25, and 50 shots is highlighted in bold.

Approach	ResNet18/(Shots)			ResNet34/(Shots)			ResNet50/(Shots)		
	5	25	50	5	25	50	5	25	50
Baseline	38.766	25.88	23.08	36.7	24.5	21.14	**34.28**	**22.56**	**19.44**
Baseline++	47.38	36.38	33.34	45.5	36.24	33.46	49.42	35.28	30.76
Triplet Network	47.22	31.18	26.08	47.06	29.48	24.58	50.72	31.26	25.3
DAML	42.54	28.56	23.86	42.6	27.12	22.2	45.78	29.3	25.34

**Table 8:** Comparison of the baseline model (the best performing model), Triplet network (ResNet50), and Triplet network Argueso et al. [38].

	5-Shots	25-Shots	50-Shots
Baseline (ResNet50)	85.58%	96.02%	97.52%
Triplet Network (ResNet50)	81.86%	93.26%	95.20%
Argueso et al. [38]	72%	85%	88%

## Data Availability

Data is available from its original source as cited in the article. The PlantVillage dataset is available at https://github.com/spMohanty/PlantVillage-Dataset and the Coffee dataset at https://github.com/esgario/lara2018/.
